# Exosomal lncRNAs as diagnostic and therapeutic targets in multiple myeloma

**DOI:** 10.3389/fonc.2024.1522491

**Published:** 2025-01-16

**Authors:** Hong Yan, Nan Jiang, Xiaoying Li, Chenyang Lin, Fang Wang, Juan Zhang, Lijuan Chen, Dan Li

**Affiliations:** ^1^ School of Laboratory Medicine, Chengdu Medical College, Chengdu, Sichuan, China; ^2^ School of Dental Medicine, Dalian University, Dalian, Liaoning, China; ^3^ Department of Laboratory Medicine and Sichuan Provincial Key Laboratory for Human Disease Gene Study, Sichuan Provincial People’s Hospital, University of Electronic Science and Technology of China, Chengdu, China; ^4^ Department of Hematopathology, The Second Affiliated Hospital of Chengdu Medical College, China National Nuclear Corporation 416 Hospital, Chengdu, Sichuan, China

**Keywords:** lncRNA, ceRNA, exosomes, multiple myeloma, biological markers, dsRNA, RNA binding proteins

## Abstract

Multiple Myeloma (MM) is the second most common malignancy of the hematopoietic system, accounting for approximately 10% of all hematological malignancies, and currently, there is no complete cure. Existing research indicates that exosomal long non-coding RNAs (lncRNAs) play a crucial regulatory role in the initiation and progression of tumors, involving various interactions such as lncRNA-miRNA, lncRNA-mRNA, and lncRNA-RNA binding proteins (RBP). Despite the significant clinical application potential of exosomal lncRNAs, research in this area still faces challenges due to their low abundance and technical limitations. To our knowledge, this review is the first to comprehensively integrate and elucidate the three mechanisms of action of exosomal lncRNAs in MM, and to propose potential therapeutic targets and clinical cases based on these mechanisms. We highlight the latest advancements in the potential of exosomal lncRNAs as biomarkers and therapeutic targets, offering not only a comprehensive analysis of the role of exosomal lncRNAs in MM but also new perspectives and methods for future clinical diagnosis and treatment of multiple myeloma.

## 1 Introduction

Multiple myeloma (MM) predominantly affects middle-aged and elderly populations and is characterized by malignant proliferation of bone marrow plasma cells and abnormal secretion of immunoglobulins and their peptide chains (1). It ranks second among hematologic malignancies, comprising approximately 10% of all hematologic tumors in high-income countries (2). MM-associated syndromes include recurrent infections, anemia, bone pain, bone lesions, limb weakness, and kidney disease (3). Despite significant improvements in survival rates due to advances in autologous hematopoietic stem cell transplantation and the emergence of new drugs such as bortezomib, carfilzomib, lenalidomide, and pomalidomide (4), patients with MM often experience relapse, develop drug resistance post-treatment, and remain incurable. With the rapid development of novel detection technologies, such as fluorescence *in situ* hybridization, high-throughput sequencing, gene chips, and quantitative polymerase chain reaction, numerous studies have indicated the crucial role of long non-coding RNAs (lncRNAs) in the pathogenesis and progression of MM (5). Investigating the formation and evolution of myeloma from the molecular and genetic perspectives can provide a key theoretical foundation and practical value for its prevention and treatment.

Extracellular vesicles (EVs) serve as bioactive transport substances released by various cell types and play an important role in the occurrence and development of various diseases (6–8). Recent domestic and international studies have found correlations between the types and quantities of EVs and the disease status and prognosis of MM patients, providing new insights into the diagnosis, prognostic assessment, and targeted therapy of MM (9, 10). In recent years, EVs have shown promising potential in the pathogenesis, diagnosis, prognosis, and treatment of MM (10).

Long noncoding RNAs (lncRNAs) are RNAs that are longer than 200 nucleotides, possess diverse regulatory functions and are transcribed by RNA polymerase II. They undergo splicing and processing to form mature lncRNAs, which, however, are not translated into proteins. lncRNAs regulate genes at the epigenetic, transcriptional, and post-transcriptional levels (11, 12). Increasing evidence suggests that dysregulation of lncRNA function is implicated in various diseases, particularly cancer (5, 13). LncRNAs in EVs associated with MM primarily regulate the expression of genes and proteins within tumor cells through mechanisms such as “competitive endogenous RNA (ceRNA)”, “RNA duplex,” and “lncRNA-RNA-binding proteins (RBP),” thereby affecting the occurrence, development, and drug resistance of MM.

In recent years, there has been a plethora of research on the role of extracellular vesicles (EVs) in multiple myeloma (MM); however, the majority of studies have focused on microRNAs (miRNAs) within EVs, with long non-coding RNAs (lncRNAs) constituting only a small fraction. Yet, due to their sheer number, diverse regulatory mechanisms, and significant functionality, lncRNAs hold the potential to become the largest repository of targets for the development of gene therapy drugs. Moreover, although some exosomal lncRNAs associated with tumor development have been identified, there are currently no anticancer drugs targeting these exosomal lncRNAs on the market. Additionally, membrane vesicles derived from cells, including exosomes and microvesicles, are considered ideal delivery systems due to their low antigenicity, low cytotoxicity, and ability to bypass endocytic pathways and phagocytosis. The biodistribution of these membrane vesicles can be tailored through their specific composition and cells of origin to meet particular needs. Therefore, there is an urgent need to identify exosomal lncRNAs associated with disease biology, to delve into their cellular functions and biological mechanisms, in order to establish their basis as therapeutic targets for multiple myeloma. This review aims to summarize the mechanisms and clinical significance of exosomal lncRNAs in MM, emphasizing their mechanisms and the latest advancements, and to reveal their potential as effective therapeutic targets and diagnostic biomarkers, in the hope of providing new therapeutic strategies and clinical applications for MM.

## 2 Role of exosomes in the pathogenesis of MM

EVs play crucial roles in MM pathogenesis and have received significant attention in cancer research. Recent studies revealed various aspects of EV biogenesis, regulation, and function in cancer cells. Han et al. (14) have provided a comprehensive overview of EV biogenesis mechanisms, emphasizing the therapeutic significance of targeting EV biogenesis in cancer treatment. Guo et al. (15) focused on the impact of EVs on the formation of premetastatic niches in tumors, highlighting their effects on inflammation, immune responses, and angiogenesis. Yang et al. (16) explored the link between EVs and metabolic reprogramming in tumors and offered new insights into cancer prevention and treatment. Additionally, Paskeh et al. (17) discussed the novel roles of EVs in cancer progression and reshaping the tumor microenvironment, emphasizing their therapeutic value in cancer treatment. Zhang et al. (18) have designed neutrophil-derived EV-like vesicles for targeted cancer therapy, providing a unique approach for precise cancer treatment using EVs. Collectively, research advancements in tumor-derived EVs indicate that these EVs play multifaceted roles in cancer progression, microenvironment remodeling, and targeted therapy. These findings collectively enhance our understanding of the complex interactions between EVs and tumors, and pave the way for innovative strategies for cancer diagnosis and treatment.

Numerous studies have demonstrated that EVs released by the host or cancer cells are involved in the initiation, growth, infiltration, and metastasis of tumors. Furthermore, EVs play a dual role in the communication between immune and cancer cells by regulating tumor immune responses. EVs contain proteins, cytokines, lipids, miRNAs, lncRNAs, and circular RNAs (circRNAs) that play crucial roles in intercellular communication and are involved in various physiological and pathological processes (10, 19). Therefore, EVs play significant roles in the pathogenesis of MM and serve as novel biomarkers and therapeutic tools in MM (20). Different types of cells can release different types of EVs that play important roles in the occurrence and development of various diseases, with varying levels in patients with MM and healthy individuals. Roccaro et al. demonstrated that BMSC-EXs derived from patients with MM promote tumor growth, whereas BMSC-EXs obtained from healthy donors inhibit MM cell proliferation (21, 22). Wang et al. (23) demonstrated that EVs derived from bone marrow adipocytes lead to MM drug resistance by inhibiting chemotherapy-induced tumor cell apoptosis. Liu et al. (24) showed that EVs from MM cells inhibit osteoblast differentiation and enhance IL-6 secretion, leading to poor bone health. Additionally, Frassanito et al. (25) emphasized the role of MM cell-mediated, EV-mediated miRNA regulation in altering the bone marrow microenvironment and affecting disease progression. Long et al. (26) demonstrated *in vitro* experiments using cell lines that BMSC-derived EV miR-182 can be transferred to MM cells to regulate their proliferation, metastasis, and resistance to carfilzomib. Sun et al. (27) reported that cancer-associated fibroblast (CAFs)-derived EV miR-21 entering MM endothelial cells (MMECs) promotes MM proliferation, invasion, and vascular formation. Liu et al. (28) revealed that C6-ceramide treatment inhibited the proangiogenic activity of MM EVs, providing insights into potential therapeutic strategies targeting the EV pathway. EVs have a significant impact on the pathogenesis of MM, owing to their influence on the bone marrow microenvironment and their involvement in regulating immune responses. Understanding the complex interactions of EVs in MM may facilitate the development of new diagnostic and therapeutic strategies.

## 3 Role of lncRNA in the pathogenesis of MM

Recent studies have shown that lncRNAs participate in the occurrence, progression, and metastasis of tumors (29). First, lncRNAs can regulate the gene expression by interacting with other RNAs such as mRNA and miRNAs, among which lncRNAs act as large “sponges” that bind to miRNAs and form ceRNA networks (30, 31). In this network, lncRNAs attract miRNAs, reducing the available concentration of miRNAs and thereby decreasing miRNA binding to the target mRNA, ultimately leading to increased stability of the target mRNA and higher transcriptional levels. Second, lncRNAs can interact with proteins or DNA, affecting the chromatin structure and transcriptional regulation (32, 33). lncRNAs regulate the chromatin status by interacting with chromatin-modifying enzymes or transcription factors, thereby influencing the target gene expression. Additionally, lncRNAs can serve as molecular bridges, regulate protein-protein interactions, and affect cell signaling and gene expression (33). These modes of action indicate that lncRNAs participate in key processes of tumor cell transformation, proliferation, apoptosis, and migration in multiple ways. We have organized the lncRNAs mentioned in this article that affect the occurrence and development of multiple myeloma into a table, as shown in **Table 1**.

**Table 1:** Mechanisms and targets of exosomal long non-coding RNAs in multiple myeloma.

LncRNA	Mechanism	Target/Pathway	Function	Donor Cells	Recipient Cells	Reference
MALAT1	ceRNA	miR-1271-5p miR-15a/16	Proliferation↑ Angiogenesis↑	Unknown Unknown	MM Cells MM Cells	(35) (36)
H19	ceRNA	miR-29b-3p miR-532-3p	Drug resistance↑ Osteogenic differentiation↓	Unknown Unknown	MM Cells MM Cells	(38, 39) (40)
NEAT1	ceRNA RBP	miR-214 EZH2	TME remodeling Immune escape	MM Cells MM Cells	NK cells NK cells	(42) (85, 86)
LINC01003	ceRNA	miR-33a-5p	Apoptosis↓	Unknown	MM Cells	(43)
OIP5-AS1	ceRNA	miR-410	Proliferation↑ Apoptosis↓	Unknown	MM Cells	(44)
LINC00461	ceRNA	miR-15a/16	Proliferation↑ Angiogenesis↑	MSCs	MM Cells	(49)
RUNX2-AS1	duplexes ceRNA	pre-mRNA miR-6797-5p	Osteogenic differentiation↓ Osteogenic differentiation↑	MM Cells MM Cells	MSCs MSCs	(72) (74)
PSMA3-AS1	duplexes	pre-mRNA	Drug resistance↑	MSCs	MM Cells	(75–77)
LOC606724	RBP	METTL7A	Apoptosis↓	Adipocytes	MM Cells	(89, 90)

"↑" represents a promoting effect, "↓" represents an inhibitory effect.

lncRNAs promote the occurrence and development of MM by promoting MM cell proliferation and invasion, maintaining the cell cycle, drug resistance, inhibiting osteogenesis, and reshaping the TME. lncRNAs can directly mediate protein expression, activate signaling pathways, or act as ceRNAs to regulate the miRNA expression by acting on downstream genes, thereby promoting the occurrence and development of MM (34). Conversely, lncRNAs inhibit the occurrence and development of MM by inhibiting the MM cell proliferation and cell cycle arrest and promoting cell apoptosis.

Recent studies have shown that lncRNAs can directly promote the proliferation, invasion, and regulation of the cell cycle, inhibition of bone formation, drug resistance, and TME remodeling of MM tumor cells by targeting miRNAs, thereby promoting the occurrence and development of MM and leading to a poor prognosis. Liu et al. (35) demonstrated that MALAT1 regulates the expression of SOX13 by targeting miR-1271-5p through a ceRNA mechanism, thereby promoting MM cell proliferation, invasion, and glycolytic ability. In addition, lncRNA MALAT1 is highly expressed in MM and acts as a competitive endogenous RNA for microRNA-15a/16 to promote the expression of vascular endothelial growth factor A (VEGFA), facilitating angiogenesis in MM (36). Shen et al. (37) demonstrated that knockdown of lncRNA AL928768.3 significantly inhibited the MM cell proliferation and colony formation, induced MM cell cycle arrest in the G0/G1 phase, inhibited the expression of CDK2 and CCND1, and promoted P21 expression. Additionally, lncRNA H19 is reported to significantly upregulate in the serum of MM patients. H19 indirectly regulates the MCL-1/Akt pathway by targeting miR-29b-3p to induce MM resistance to bortezomib or doxorubicin (38, 39). In a recent study, H19 was shown to act as a sponge for miR-532-3p to upregulate E2F7 and inhibit tumor suppressor gene (PTEN) epigenetics. Guo et al. (40) concluded after *in vivo* experiments that H19 disrupts the balance between osteogenesis and osteolysis through Akt/mTOR signal transduction, leading to reduced osteogenic activity and increased osteoclast activity, promoting the occurrence and development of MM. Additionally, EV-derived lncRNA RUNX2-AS1 can be delivered to mesenchymal stem cells (MSCs) through vesicle transfer, inhibiting their osteogenic activity (41). lncRNAs can also participate in the remodeling of the TME in MM cells. For example, lncRNA NEAT1, highly expressed, inhibits miR-214, upregulates the expression and release of B7-H3, promotes M2 macrophage polarization, and accelerates MM progression (42).

**Figure 1:**
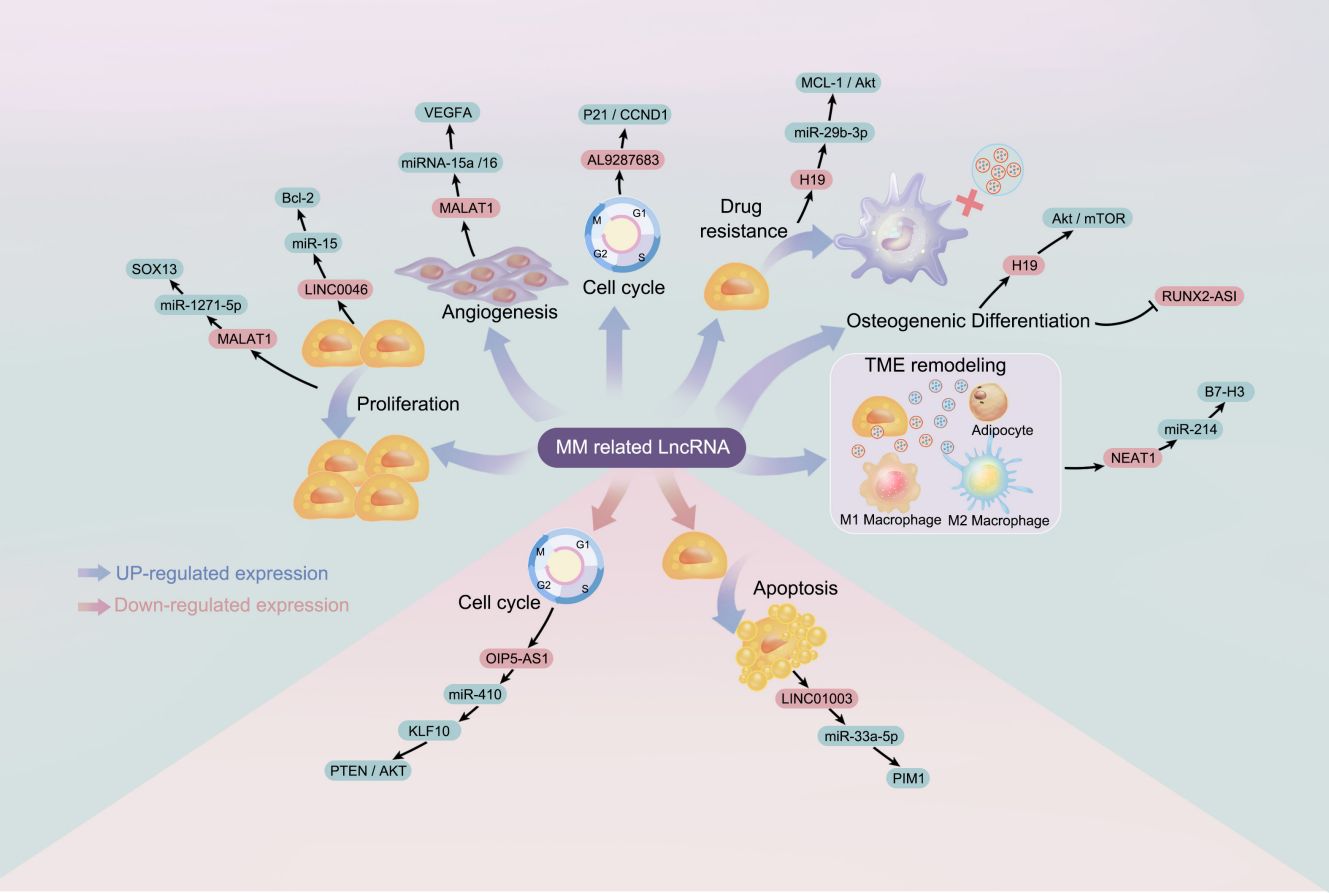
The regulatory role of long non-coding RNA (lncRNA) in the pathogenesis of multiple myeloma: lncRNA refers to long non-coding RNA; miRNA refers to microRNA.

Few studies have shown that lncRNAs can also inhibit the occurrence and development of MM through various pathways, such as cell cycle arrest and the promotion of cell apoptosis. Using the dual-luciferase reporter gene method, Wu et al. (43) demonstrated that LINC01003 inhibits the MM cell vitality and adhesion by upregulating the expression of the miR-33a-5p target gene, PIM1, as a sponge for miR-33a-5p, promoting MM cell apoptosis. In addition, OIP5-AS1 negatively regulates miR-410, and miR-410 further directly targets KLF10, thereby negatively regulating KLF10 expression. KLF10 mediates the PTEN/AKT signaling pathway, forming the OIP5-AS1-miR-410-KLF10/PTEN/AKT signaling axis. Compared to normal tissues, the lncRNA OIP5-AS1 is downregulated in MM tissues and miR-410 expression is upregulated, promoting MM cell proliferation, cell cycle progression, and inhibition of cell apoptosis (44). In addition, Jiang et al. (45) downregulated the expression of the lncRNA IRAIN *in vitro*, promoting MM cell proliferation, and revealing its potential as a new therapeutic target for MM. These lncRNAs are expected to become new targets and biomarkers for MM treatment with broad prospects for cancer treatment, as shown in **Figure 1**.

## 4 Role of exosomal lncRNA in MM

Exosomes associated with MM mostly carry proteins and RNA, including non-coding genetic material, which are closely related to various biological processes involved in the disease. Currently, miRNAs are the most extensively studied substances in exosomes. However, in recent years, there has been growing interest in the lncRNA, both domestically and internationally, especially in the field of MM. lncRNAs are excellent biomarkers, and their detection in exosomes may have significant implications for patients with MM. In addition, exosomes can be viewed as potential drug carriers and are promising candidates for clinical therapies.

The role of lncRNAs in the pathogenesis of MM has attracted increasing attention because of their potential impact on the disease process. Exosomal lncRNAs have been found to be involved in various key mechanisms of MM, such as drug resistance, immune escape, and tumor progression (23, 41, 46). For instance, Wang et al. elucidated the inducing effect of m6A methylation in adipocyte-derived exosomal lncRNAs on drug resistance in MM, providing insights into previously unexplored mechanisms exacerbating MM drug resistance and suggesting potential strategies to enhance therapeutic efficacy (23). Their work also revealed the role of the exosomal lncRNA NEAT1 in inhibiting NK cell activity and promoting the immune escape of MM cells, offering a new therapeutic potential for MM by targeting exosomal lncRNAs (47). Long noncoding RNA 00461 (LINC00461) is an important member of the lncRNA family that plays a role in the occurrence and development of various cancers. Knockdown of LINC00461 enhances the antitumor effects of dexamethasone and promotes apoptosis in MM cells. Similar effects were observed in studies involving MSCs, and exosomes derived from MSCs promoted MM progression of MM, indicating a possible association with LINC00461 (48). Furthermore, other studies have demonstrated the broader relevance of exosomal lncRNAs in cancer biology and clinical management, as exosomal lncRNAs promote proliferation, metastasis, and acquisition of drug resistance in various cancers, including MM (36, 49). These findings collectively underscore the importance of exosomal lncRNAs in the pathogenesis of MM and highlight their potential as diagnostic markers and therapeutic targets for disease management. Building on this foundation, further analyses and discussions on the mechanisms of action of exosomal lncRNAs in MM will be conducted.

### 4.1 “ceRNA”

**Figure 2:**
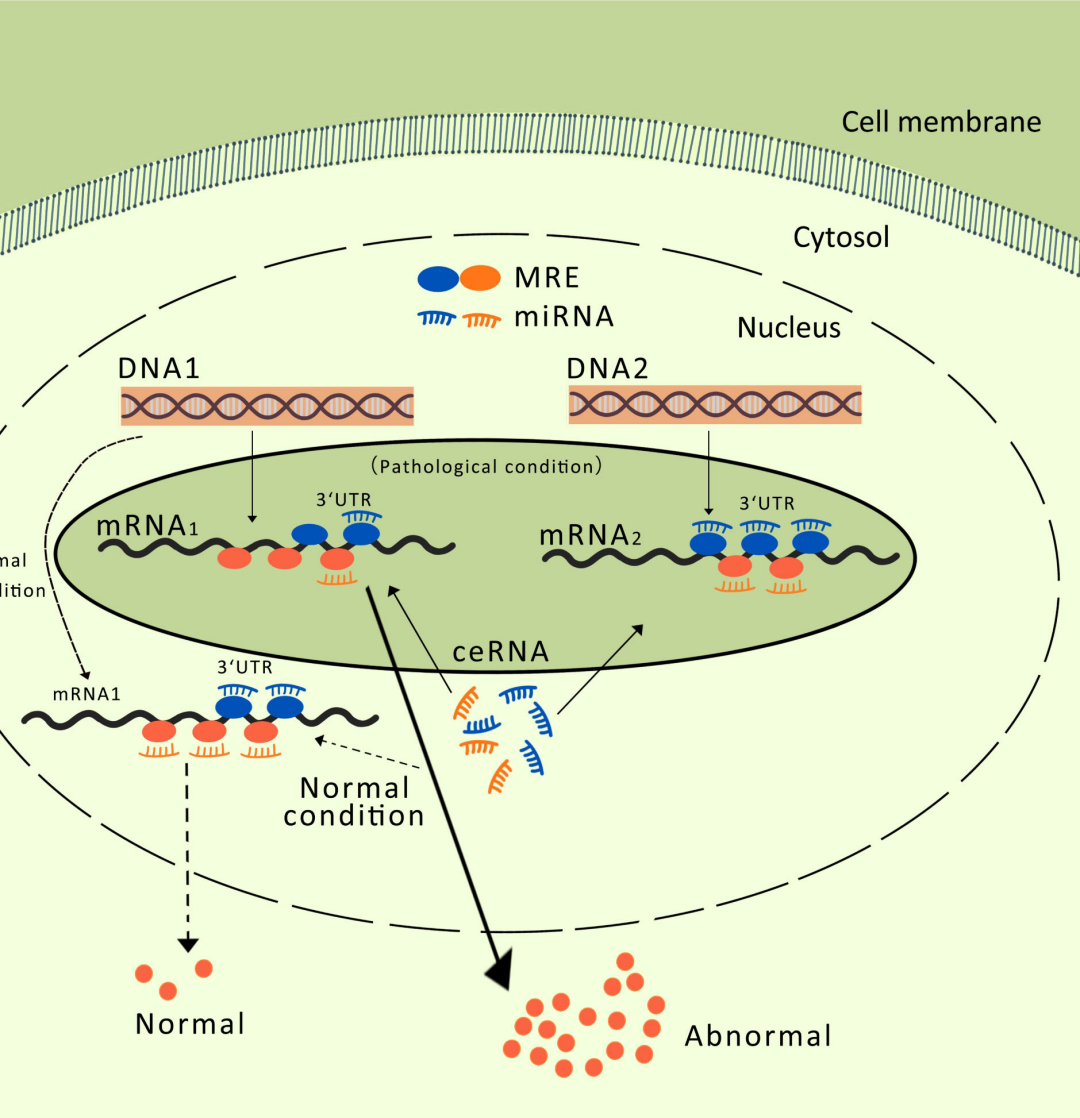
MREs, miRNA Response Elements. The competitive endogenous RNA mechanism: When an RNA2 molecule binds miRNA through a miRNA binding site, it can prevent miRNA from binding to other RNAs that share the same binding site, thereby reducing the inhibitory effect of miRNA on those other RNAs (such as RNA1), resulting in an increased expression level of these other RNAs (RNA1). This interaction, regulated by miRNA, creates a complex regulatory network that impacts the balance and regulation of gene expression.

ceRNA networks have attracted extensive research interest in various cancers, including gastric cancer, intervertebral disc degeneration, epithelial ovarian cancer, cervical cancer, and hepatocellular carcinoma (50–54). Different RNA transcripts compete for miRNA response elements to regulate free miRNA expression, thereby facilitating mutual regulation. lncRNAs and miRNAs are interrelated in cancer, and lncRNA-mRNA-miRNA networks have been constructed in MM (30, 55–60). These findings provide new hope for the diagnosis, prognosis, and treatment of MM, and pave the way for the development of precision medicine. lncRNAs regulate MM progression through a ceRNA mechanism, demonstrating their potential clinical applications and biomarker values (36). Exosomal lncRNAs are considered therapeutic targets and biomarkers for malignant tumors, aiding in diagnosis, prognostic assessment, and drug treatment research. The ceRNA mechanism is illustrated in **Figure 2**.

Exosomal LINC00461 promotes the proliferation of MM cells. MSCs, a major member of the stem cell family, play crucial roles in tissue repair, cancer treatment, and immune regulation and are widely used in the study and treatment of various human diseases (61). Exosomal transfer of regulatory RNAs mediated by MSCs and MM cell sources is a key feature of cancer cell formation, promoting the tumor microenvironment, and regulating MM cell proliferation, spread, invasion, and drug resistance (62, 63). The role of lncRNAs in MM proliferation and progression is increasingly being recognized, with one lncRNA, LINC00461, promoting MM cell proliferation (64). LINC00461 is located in the intergenic region between the human chromosome 5 protein-coding genes MEF2C and TMEM161B. It is a ceRNA associated with 18 miRNAs and is overexpressed in respiratory, digestive, urinary, nervous system, and hematological malignancies (65). Deng et al. (49) demonstrated that the expression of LINC00461 in plasma cells from patients with MM was significantly higher in MSCs from adjacent tissues than in those from the control group. Moreover, LINC00461 is enriched in exosomes derived from MSCs of adjacent tissues and transferred to MM cells, affecting their proliferative ability. In gene knockout experiments, LINC00461 knockdown resulted in MM cell apoptosis and G1 cell cycle arrest, significantly inhibiting cell proliferation and migration. Overall, MSC-derived LINC00461 plays an important role in regulating MM cell proliferation, and its high expression significantly correlates with patient prognosis and poor survival.

LINC00461 acts as a ceRNA to regulate the miRNA expression. RNA structure analysis showed that miR-15a and miR-16 had two binding sites for each phenotype of LINC00461. In MS2-RIP radiolabeling immunoprecipitation assays, compared to the empty vector MS2, miR-15a/16 directly bound to LINC00461 and was enriched in MS2-LINC00461. Moreover, in cells overexpressing LINC00461, the expression levels of miR-15a and miR-16 were significantly lower than those in control group (49). In conclusion, exosomal LINC00461 has a sponge effect on miR-15a and miR-16, which is capable of binding and downregulating their expression. Zhang et al. (66) showed that low expression of miR-15a/16 in patients leads to increased expression of CABIN1 mRNA, promoting tumor proliferation. Essentially, miR-15a/16 directly targets CABIN1 mRNA and negatively regulates CABIN1 expression at both the mRNA and protein levels in MM cells. In another study, VEGFA, a target gene of miR-15a/16, affected angiogenesis in MM by regulating the expression of VEGFA (67). Calin et al. (68) showed that miR-15a/16, a natural antisense transcripts of BCL2, directly interact with BCL2 and negatively regulates its expression at the transcriptional level. BCL2, as an important gene that regulates tumor cell apoptosis, plays a crucial role in mediating MM cell apoptosis and drug resistance.

In summary, exosomal LINC00461 has a sponge effect on miR-15a/16, affecting the expression of downstream genes such as CABIN1 mRNA, VEGFA, and BCL2, and forming a ceRNA regulatory network centered on LINC00461-miR-15a/16, thereby influencing MM proliferation, invasion, and apoptosis. The ceRNA network centered on exosomal LINC00461 is still in the early stages of research, and its specific relationship with MM has not yet been elucidated. The interaction between LINC00461-miR-15a/16 may be influenced by other molecular stress conditions, making it difficult to elucidate the intrinsic regulatory mechanisms of LINC00461. In conclusion, the complex interaction between lncRNAs and MM cell proliferation emphasizes the importance of further studies on the regulatory roles of these ncRNAs in the pathogenesis of MM. Understanding the mechanism by which lncRNAs such as LINC00461 promote MM cell proliferation may pave the way for the development of new therapeutic interventions against this hematological malignancy.

### 4.2 RNA duplexes

**Figure 3:**
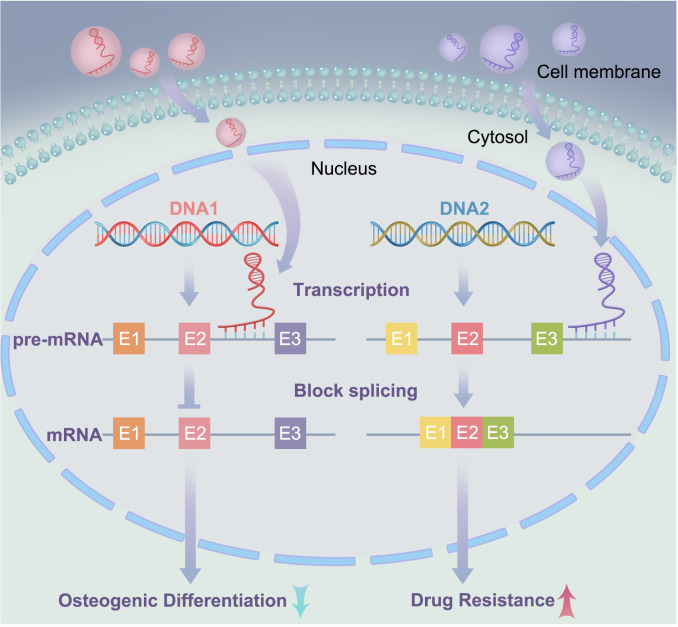
IncRNA forms RNA duplexes with mRNA: In the left signaling pathway, exosomal lncRNA binds to pre-mRNA through base complementary pairing, forming RNA duplexes that block alternative splicing of mRNA, thereby reducing the osteogenic potential of mesenchymal stem cells (MSCs); In the right signaling pathway, exosomal lncRNA forms RNA duplexes with pre-mRNA through base complementary pairing, enhancing the stability of mRNA. E1, E2, and E3 represent exons.

Extensive studies have demonstrated the complex role of exosomal lncRNAs in MM. In addition to the ceRNA mechanism mentioned above, lncRNAs can bind to mRNA to form RNA duplexes. The molecular interactions of lncRNAs play crucial roles in tumors (69, 70). Understanding the role of RNA duplexes in MM is key to understanding the molecular mechanisms underlying this complex disease. In MM, RNA duplexes play important roles in regulating gene expression, cell signaling, and intercellular communication. Understanding the various roles of exosomal lncRNAs in MM, from intercellular communication to targeted therapy through the formation of RNA duplexes with transcripts, will contribute to a better understanding of the molecular mechanisms underlying the occurrence and progression of MM. The lncRNA lncRUNX2-AS1 formed an RNA duplex with the precursor mRNA of RUNX2, reducing the osteogenic potential of MSCs (**Figure 3**). The exosome-mediated transfer of lncRUNX2-AS1 from MM cells to MSCs has been identified as a potential mechanism for inhibiting osteogenesis. Li et al. (41) demonstrated that exosomes derived from U266 or MM1S MM cells significantly reduced bone nodule formation in cocultured MSCs. Exosomes derived from MM cells with knocked-down RUNX2-AS1 reduced the intracellular levels of RUNX2-AS1 in MSCs and diminished their osteogenic capacity. Therefore, high levels of RUNX2-AS1 in MSCs were significantly correlated with the osteogenic activity. Antisense lncRNA can bind to mRNA through complementary base pairing, affecting the mRNA alternative splicing (71). Fluorescence *in situ* hybridization and reverse transcription PCR showed gene overlap between RUNX2 and RUNX2-AS1, but in opposite transcriptional directions. Overexpression of RUNX2-AS1 in MM cells significantly reduced the RUNX2/pre-RUNX2 ratio. After RNase protection experiments on RNA in bone marrow stromal cells, followed by reverse transcription PCR probing for the products, it was found that the overlapping portion of the transcripts was protected and not degraded (41). Therefore, RUNX-2AS1 acts as a highly specific sensor for mRNA and forms an RNA duplex with RUNX2 through overlapping base pairs. This interaction blocks the splicing of the RUNX2 pre-mRNA, thereby negatively regulating RUNX2 mRNA. Unlike the lncRNA-miRNA axis, RUNX2 is a direct target gene of RUNX2-AS1, which directly regulates osteoblast differentiation and indirectly regulates osteoclast differentiation (72). In mouse models, Xu et al. (73) found that osteoblasts lacking RUNX2 produce a highly chemoattractive and immunosuppressive bone marrow microenvironment, affecting the localization and progression of MM to new bone sites. In summary, the exosomal lncRNA RUNX2-AS1 derived from MM cells acts as a natural antisense transcript of RUNX2 and directly binds to and downregulates the expression of RUNX2 in bone marrow stromal cells, thereby inhibiting bone formation.

Furthermore, in studies by Arumugam et al. (74), the lncRNA RUNX2-AS1 also had a sponge effect on miR-6797-5p, forming the RUNX2-AS1/miR-6797-5p/RUNX2 axis, negatively regulating the expression of RUNX2, and promoting osteoblast differentiation. Most studies reporting ceRNA communication between RNA molecules have focused on binary interactions between two transcripts, but these interactions are mediated by one or more miRNAs. The two regulatory modes of the exosomal lncRNA RUNX2-AS1 illustrate the complexity of the relationships between the components in ceRNA regulatory networks.

The lncRNA, lncPSMA3-AS1, forms an RNA duplex with the precursor mRNA of PSMA3, promoting resistance to proteasome inhibitors in MM. In previous studies, lncRNA PSMA3-AS1 was shown to regulate the expression of corresponding miRNAs in various tumors, such as cholangiocarcinoma, gliomas, and pancreatic ductal adenocarcinoma (75–77). Xu et al. (78) showed that the mRNA levels of PSMA3 and PSMA3-AS1 in CD138+ MM cells were significantly elevated in patients with bortezomib-resistant MM compared with those in patients with bortezomib-sensitive MM. PSMA3-AS1 is mainly encapsulated in MSC-derived exosomes, transferred to MM cells, and enriched therein, further affecting their resistance to proteasome inhibitors. Kaplan-Meier analysis showed that high levels of PSMA3 in CD138+ MM cells correlated with decreased progression-free survival and overall survival in patients with MM, and high levels of PSMA3 could also serve as an independent prognostic factor for patients with MM receiving bortezomib treatment. All the above findings indicate a correlation between PSMA3-AS1 and disease progression and proteasome inhibitor resistance in patients with MM.

The target gene of PSMA3-AS1 is the sense transcript PSMA3. Fluorescence *in situ* hybridization and nuclear-cytoplasmic fractionation analyses showed that PSMA3-AS1 coexists in both the nucleus and cytoplasm. PSMA3 and PSMA3-AS1 are located on chromosome 14p23.1, and PSMA3-AS1 overlaps with intron 7 of PSMA3 by 2029 nucleotides. Previous studies have shown that antisense transcripts can regulate the sense transcript in two ways: first, by positively regulating the expression of the sense transcript, and second, by negatively regulating its expression (79). After blocking new RNA synthesis with α-amanitin, the loss of PSMA3, PSMA3-AS1, GAPDH, and 18s RNA was measured. The results showed that knocking out PSMA3-AS1 reduced the stability of PSMA3, whereas PSMA3-AS1 overexpression increased the stability of PSMA3. This finding suggests that PSMA3-AS1 can form a duplex with PSMA3, increasing its stability of PSMA3 by reducing its degradation and thereby positively regulating the expression of PSMA3 (78).

Previous studies have shown that PSMA3 encodes the alpha 7 subunit of the proteasome, which plays an important role in its formation and function (80). PSMA3 and PSMA3-AS1 knockout resulted in decreased proteasome activity in MM cells, whereas overexpression increased proteasome activity. In summary, PSMA3-AS1 can bind to PSMA3-AS1 pre-mRNA and increase its stability, promoting the proliferation of MM cells and proteasome activity, and conferring resistance to proteasome inhibitors in MM cells, as shown in **Figure 3**. *In vivo* experiments by Xu et al. (78) showed that exogenously injected therapeutic PSMA3-AS1 siRNA effectively increases the sensitivity of U266 xenografts to carfilzomib, significantly prolonging overall survival when combined with carfilzomib treatment. These experiments further demonstrate how the biological activity of the exosomal lncRNA PSMA3-AS1 can be transmitted between different cell types, affecting cell function, and may serve as a therapeutic target for MM bone lesions. RNA-based therapies, particularly siRNA, have shown great potential in cancer treatment because they can silence key genes in tumor progression (81). *In vivo* siRNA delivery can be achieved through various methods such as liposomes, lipid nanoparticles, polymeric nanoparticles, viral vectors, proteins and peptides, and exosomes, to enhance the stability of siRNA and cellular uptake efficiency, and to overcome delivery barriers (82). Preclinical and clinical studies have demonstrated the potential for treating solid tumors and hematological malignancies, as well as cancer immunotherapy. This indicates a novel signaling pathway involved in drug resistance and highlights the role of exosomes in intercellular communication in the context of this disease.

### 4.3 IncRNA RBP

Complex interactions between exosomal lncRNAs and RBPs have garnered significant attention. Exosomes are EVs involved in intercellular communication and are associated with the progression and drug resistance of MM (83). RBPs have been identified as promising biomarkers for MM and the construction of RBP signatures can effectively predict the prognosis of patients with MM. The interaction between lncRNAs and RBPs plays a crucial role in the occurrence and development of cancer (84–86), such as regulating target gene expression, RBP activity and stability, and lncRNA expression levels (11). These findings emphasize the importance of further exploration of these molecular mechanisms to develop new therapeutic strategies and improve patient prognosis.

**Figure 4:**
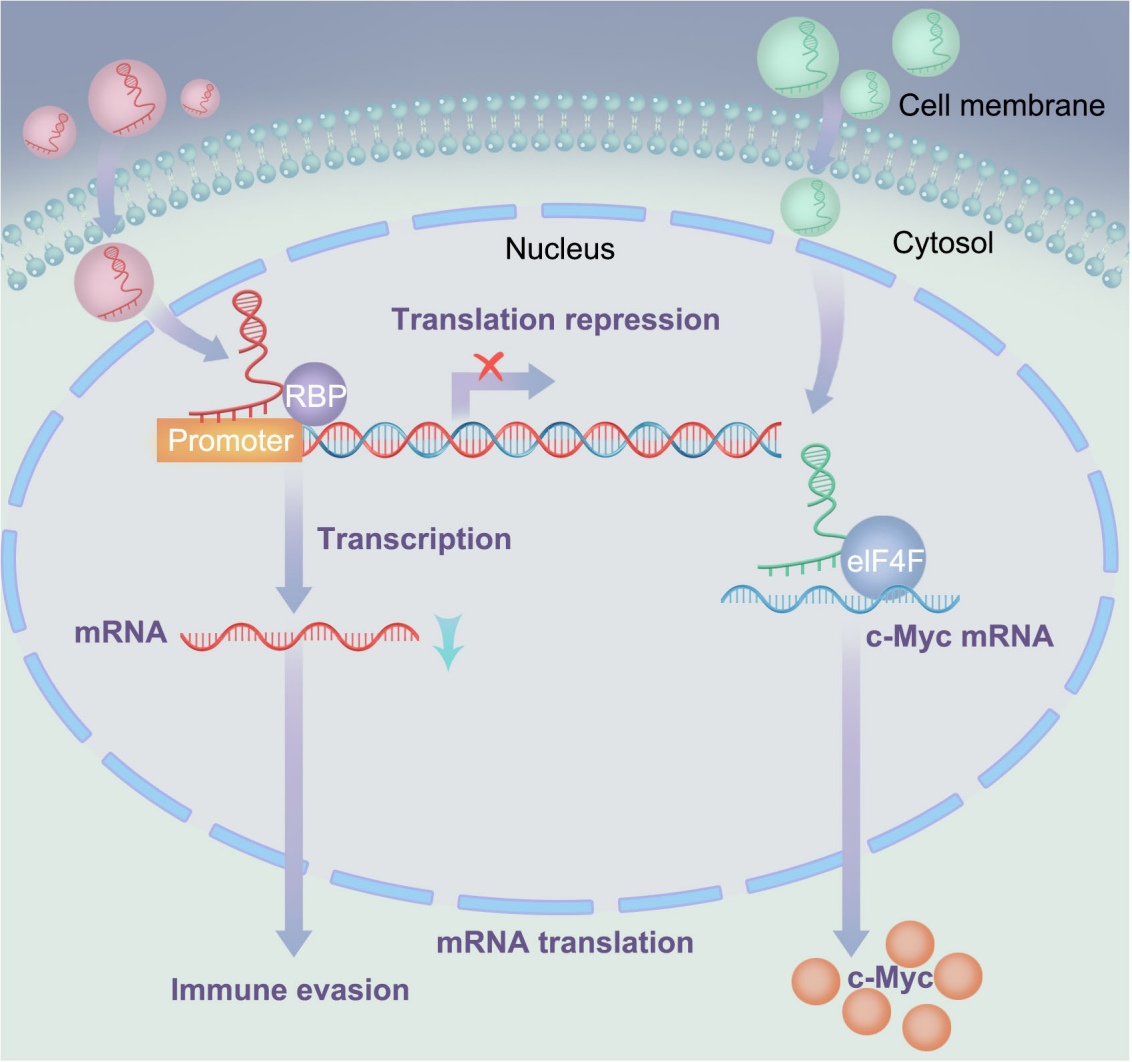
Interactions between lncRNA and RNA-binding proteins: In the left signaling pathway, lncRNA suppresses the expression of downstream effectors (mRNA) by recruiting RNAbinding proteins; In the right signaling pathway, LOC606724 acts as a bridge connecting eIF4E and c-Myc, upregulating c-Myc protein at the post-transcriptional level in MM cells.

Exosomal lncRNA NEAT1 binds to EZH2 and promotes immune escape of MM cells. NEAT1 is a long non-coding RNA that binds to EZH2 and regulates the expression of its downstream effectors (87, 88). EZH2 is a histone methyltransferase involved in the occurrence and development of various tumors (89, 90). The interaction between NEAT1 and EZH2 enables EZH2 to bind to the promoter region of PBX1, thereby inhibiting PBX1 expression through H3K27 trimethylation, as shown in **Figure 4**. Studies have shown that NEAT1 can affect EZH2 modification of target gene promoter regions by interacting with EZH2, thereby regulating gene expression (47). In this study, NEAT1 inhibited PBX1 expression by recruiting EZH2, thereby suppressing NK cell activity and promoting the immune escape of MM cells. Natural killer (NK) cells are important immune cells with cytotoxic and inhibitory activities against cancer cells. However, MM cells can evade immune surveillance by inhibiting NK cell activity. These findings provide new strategies for the treatment of MM, and exosomal lncRNA NEAT1 has the potential to become a new therapeutic target for MM.

Exosomal LOC606724 binds to METTL7A and inhibits apoptosis in MM cells. In current research, lncRNAs play important regulatory roles in the interaction between MM cells and adipocytes, with adipocytes promoting obesity-induced myeloma (91, 92). Studies have also identified interactions between lncRNAs and RNA-binding proteins and identified METTL7A as an RNA methyltransferase. Further experiments showed that MM cells promoted the packaging of lncRNAs into adipocyte-derived exosomes via METTL7A-mediated lncRNA m6A methylation. Previous experiments have demonstrated that MM cells can reprogram adipocytes through direct intercellular interactions, upregulating EZH2 expression and activating histone methylation in adipogenic factor promoters (93). This suggests that the methylation modifications of lncRNAs play an important regulatory role in MM. Conversely, adipocyte-derived exosomes protect MM cells from chemotherapy-induced apoptosis. Wang et al. (23) showed that two lncRNAs, LOC606724 and SNHG1, were significantly upregulated in MM cells exposed to adipocyte-derived exosomes. LOC606724, as a bridge connecting eIf4E and c-Myc, upregulated the c-Myc protein in MM cells at the post-transcriptional level (**Figure 4**). This indicates the existence of a malignant cycle between MM cells and adipocytes. Blocking this exosome-mediated malignant cycle may be a potential strategy to improve treatment efficacy.

In summary, exosomal lncRNAs have multiple mechanisms in MM, including the regulation of cell apoptosis, methylation modifications, and protein transformation. These findings provide important clues for a deeper understanding of MM pathogenesis, and offer potential targets for developing new therapeutic strategies. However, further research is needed to validate these findings and explore the association between lncRNAs and MM, which will greatly assist in devising more effective treatment strategies for patients with MM.

## 5 Exosomal lncRNAs as therapeutic targets in MM: clinical implications

Currently, research on exosomal lncRNA in multiple myeloma (MM) is still in its infancy, and as a novel diagnostic and therapeutic approach, it faces several challenges. Firstly, the techniques for exosome isolation and purification vary at present, and the yield of native exosomes is too low to meet the demands of clinical applications. It is crucial to optimize and upgrade the isolation and purification technologies for exosomes to enhance their purity and yield (94). Secondly, the mechanism of action of exosomal lncRNA in MM is not yet fully understood, necessitating further basic and clinical research, as well as multicenter collaborative studies, to fully explore the role of exosomes in aiding the diagnosis, prognosis assessment, and treatment guidance of MM. Thirdly, the high heterogeneity of multiple myeloma complicates the study of the role of exosomal lncRNA in MM (95). Fourthly, the identification of biomarkers: although the abnormal cargo in exosomes can be used as cancer biomarkers for the detection or screening of early prognosis in MM patients, identifying these biomarkers remains a challenge. Combining DNA signal amplification techniques with the signal enhancement characteristics of nanomaterials offers a promising solution to address these limitations and improve the efficiency and accuracy of exosome detection (96). Fifthly, the determination of therapeutic targets: identifying exosomal lncRNA as therapeutic targets requires in-depth research into their specific roles and mechanisms in the progression of MM. In summary, we believe that exosomal lncRNA may play a significant role in the diagnosis and treatment of multiple myeloma in the future.

Compared to traditional drug delivery systems, exosomes offer outstanding biocompatibility, high specificity, and effective drug release capabilities (97–99). Loading drugs into exosomes enables precise delivery to affected sites, thereby enhancing treatment efficacy and reducing adverse reactions. Additionally, exosomes have potential as diagnostic tools because they contain rich information on biomarkers that can be utilized for early disease diagnosis and treatment prediction (100, 101). For example, circulating exosomes can serve as cancer biomarkers and aid in early detection and monitoring. Furthermore, recent studies have suggested that ceRNAs can also serve as potential therapeutic targets and biomarkers for analyzing the pathogenesis of malignant tumors (57, 102, 103), demonstrating significant clinical application and research significance. There are also abundant data suggesting that exosomal lncRNAs can serve as potential therapeutic targets for malignant tumors (104–106), aiding in diagnosis, prognosis prediction, early assessment of specific drug treatment effects, and drug resistance issues, and even indicating directions for exploring the mechanisms of malignant tumor formation.

An increasing number of studies have revealed the clinical significance of exosomal-derived lncRNAs in MM treatment, which play a key role in the diagnosis, treatment, and monitoring of various cancers (107–109). In acute myeloid leukemia (AML), extracellular lncRNAs LINC00265, LINC00467, UCA1, and SNHG1 are promising biomarkers for disease diagnosis and treatment monitoring (107). Similarly, Zhao et al. emphasized the predictive value of the lncRNA PCAT1 in patients undergoing MM-induced therapy, linking it to prognosis and treatment response (110). In MM, the interaction between exosomal lncRNAs and drug resistance mechanisms has attracted attention. Exosome-mediated SNHG16 promotes dexamethasone resistance by transferring side population cells to main population cells, revealing a new mechanism for MM exosome transfer (109). Similarly, the exosomal lncRNA NEAT1 inhibits the activity of natural killer (NK) cells, promoting MM immune escape via the EZH2/PBX1 axis (47). These findings highlight the diagnostic, prognostic, and therapeutic potential of exosomal lncRNAs in the treatment of MM. Additionally, the exploration of extracellular vesicle proteins as MM biomarkers emphasizes the development prospects of liquid biopsy to improve patient stratification and prognosis (111, 112). In summary, exosome-derived lncRNAs represent valuable targets for MM treatment and provide insights into disease monitoring, drug resistance mechanisms, and prognostic stratification. Elucidation of these molecular mechanisms holds great promise for advancing personalized treatment strategies for patients with MM and improving clinical outcomes.

## 6 Prospects and limitations

The mechanisms of action of exosomal lncRNAs are highly complex and diverse. According to current research findings, the mechanisms of exosomal lncRNAs primarily include the following aspects: epigenetic regulation, transcriptional regulation, post-transcriptional regulation (113), miRNA sponge function, molecular decoy and guide functions, scaffold functions, interactions with proteins, regulation of cellular localization, and serving as precursors to small non-coding RNAs (114). These mechanisms demonstrate the multifunctionality and importance of lncRNAs in cell biology and disease pathogenesis. They are not only involved in a variety of physiological and pathological processes but also provide new perspectives for disease diagnosis and treatment. In multiple myeloma, the interactions of lncRNA with RNA (ceRNA and RNA duplexes), lncRNA with DNA (IncRNA RBP), and lncRNA with proteins (IncRNA RBP) are more representative and play a very important role in the occurrence and development of multiple myeloma. In comparison, the mechanisms of other exosomal lncRNAs in multiple myeloma warrant further exploration.

The role of exosomal lncRNAs in multiple myeloma (MM) remains a relatively new field of study, particularly in terms of their application in the diagnosis and treatment of MM. Exosomal lncRNAs hold the potential to serve as biomarkers, which could be employed as monitoring items for the diagnosis and prognostic assessment of MM, a key aspect of personalized medicine and precision therapy. We have explored the possibility of exosomal lncRNAs as therapeutic targets, including their roles in drug resistance, immune evasion, and tumor progression, providing a theoretical foundation for the development of new treatment strategies. The review integrates the latest research findings, including new discoveries regarding exosomal lncRNAs in MM, as well as their potential applications in disease monitoring, mechanisms of drug resistance, and prognostic stratification. By analyzing the role of exosomal lncRNAs in MM from multiple perspectives, including molecular biology, genetics, and epigenetics, the review offers new insights into the field of MM research and potential new tools for clinical diagnosis and treatment.

## 7 Conclusion

Although an increasing number of genetic and epigenetic events that lead to the occurrence and development of MM have been identified, their roles in the diagnosis, treatment, and clinical outcomes of MM remain unclear. Recent studies have confirmed the involvement of lncRNA dysregulation in the transcriptional, post-transcriptional, and epigenetic regulation of MM. Multiple lncRNA-based diagnostic and prognostic models have been established (58). Compared to traditional sources of lncRNAs, exosomal lncRNAs are widely present in various body fluids and exhibit high cell and tissue specificity, making it possible to construct novel, minimally invasive biomarkers for cancer diagnosis and prognosis using exosomal lncRNAs (115, 116). Furthermore, combined with the continuously evolving ceRNA network, RNA duplexes, RNA-binding proteins, and other molecular mechanisms centered on MM, establishing reliable prediction models based on exosomal lncRNA expression characteristics, assisting in identifying new drug targets through ceRNA regulatory networks, and utilizing nucleic acid-targeted therapy to avoid MM drug resistance and disease progression will have significant implications for the diagnosis and treatment of MM patients.
